# Antireflux Surgery’s Lifespan: 20 Years After Laparoscopic Fundoplication

**DOI:** 10.1007/s11605-023-05797-4

**Published:** 2023-08-14

**Authors:** Renato Salvador, Arianna Vittori, Giovanni Capovilla, Federica Riccio, Giulia Nezi, Francesca Forattini, Luca Provenzano, Loredana Nicoletti, Lucia Moletta, Andrea Costantini, Michele Valmasoni, Mario Costantini

**Affiliations:** Department of Surgical, Oncological and Gastroenterological Sciences, University of Padua, School of Medicine, UOC Chirurgia Generale 1, Azienda Ospedale Università, Padova, Italy

**Keywords:** Laparoscopic fundoplication, GERD, Nissen, Hiatal hernia, Long-term LARS

## Background

Gastroesophageal reflux disease (GERD), with or without hiatal hernia (HH), is very common; it affects millions of individuals worldwide, with a significant economic impact and loss of health-related quality of life.^1,2^ The introduction of laparoscopic antireflux surgery (LARS) in 1991 gave patients a safe and effective alternative^3^ to medical treatment. LARS soon became widespread, almost completely replacing open fundoplication to become the surgical technique of choice for treating GERD and HH.^4^ Now that laparoscopic fundoplication is considered the leading alternative to lifelong medical antireflux therapy, it is important to assess the long-term outcome of the procedure, an aspect that has become crucial to establishing it as the best treatment option. So far, the subjective and objective short- and mid-term results of LARS have reportedly been excellent,^5–10^ but limited information is available on the longer term.

The aim of this study was therefore to examine the durability of laparoscopic fundoplication based on a follow-up of at least 20 years in a cohort of consecutive patients treated with LARS for GERD and/or large HH at a national referral center for esophageal diseases.

## Materials and methods

### Patient population

We performed a retrospective study with prospectively collecting data on all patients who underwent laparoscopic fundoplication for GERD and/or large HH at the Department of Surgical, Oncological and Gastroenterological Sciences, University of Padova (Italy), between 1992 and 2001. Patients were divided into two groups: (i) a GERD group comprising patients with abnormal exposure of the distal esophagus to gastric acid detected on pH monitoring, with or without type I sliding HH (<1/3 of the stomach herniated in the chest)^11^; and (ii) an HH group comprising patients with > 1/3 of the stomach herniated in the chest: paraesophageal (type II), mixed (type III) or complex (type IV) HH. Patients treated with concurrent Collis gastroplasty, those who had upper GI surgery for other diseases, and those who died before completing 20 years of follow-up were excluded from the study.

Patients who were routinely operated for GERD or hiatal hernia at our center were enrolled, no experimental intervention was performed, therefore the ethical approval was not deemed necessary.

### Preoperative work-up

#### GERD symptoms

GERD symptoms were recorded using the symptom score (SS) described in other studies.^12^ The severity and frequency of heartburn, acid regurgitation, chest pain, and dysphagia were scored, and the final scores were calculated by adding the severity of each symptom (0=none, 2=mild, 4=moderate, 6=severe) to its frequency (0=never, 1=occasionally, 2=once a month, 3=every week, 4=twice a week, 5=daily).

#### Endoscopy

Endoscopy was performed in all patients before surgery and intraoperatively. Esophagitis was staged according to the Los Angeles classification.^13^ Any redness and velvety texture in the esophagus were assumed to indicate non-native esophageal mucosa, but this was only classified as Barrett’s esophagus (BE) after histology (hematoxylin and eosin [H&E]) confirmed the presence of intestinal metaplasia (IM).^14^

#### Barium swallow

A barium swallow was obtained preoperatively in all patients to objectively assess, measure, and classify cases of HH (type I, sliding hernia; type II, paraesophageal hernia; type III, mixed hernia; type IV, complex hernia).^15^

#### Esophageal manometry and 24-hour pH monitoring

Esophageal manometry was performed with the conventional technologies in use at the time, using a low-compliance pneumohydraulic perfusion system (Menfis, Bologna, Italy). Details of this analysis have been reported elsewhere.^16,17^ Esophageal acid exposure was assessed with 24-hour pH monitoring. The procedure was performed in all patients at least 15 days after suspending any proton pump inhibitors (PPI) or H2-blockers. The test was used to identify any abnormal acid exposure by positioning an electrode 5 cm above the upper border of the lower esophageal sphincter (LES) according to the standard procedure adopted at our laboratory, and described elsewhere.^18,19^

### Surgical technique

The surgical technique involved: reducing the hernia (if any); primary closure of the crura with one or two stitches calibrated over a 40 French bougie; and a 360° fundoplication, sutured with three nonabsorbable stitches, including the esophageal wall in the two distal sutures. Since 1995, resection of two to four short gastric vessels to mobilize the gastric fundus and obtain a floppy, well-shaped fundoplication was routinely added to the procedure. A partial fundoplication was only performed in patients with a severely abnormal esophageal peristalsis. From 1996 onwards, a U-shaped mesh (made of PTFE on the side to be in contact with the viscera, and propylene on the side facing the crura) was used to reinforce the suture of the pillars if the hiatal defect was large (as is often the case in paraesophageal hernias).

### Postoperative assessment

Patients were examined at our outpatient clinic at 1, 6 and 12 months after undergoing LARS, then every 2 years afterwards. At each visit, heartburn, acid regurgitation, chest pain, and dysphagia were assessed with the same scoring methods as those used preoperatively, and any resumption of PPI therapy was recorded. New-onset symptoms, such as bloating, were also investigated. A barium swallow was obtained 1 month and 2 years after LARS; pathophysiological studies (esophageal manometry and 24-hour pH-monitoring) were repeated after 6 months; and endoscopy after 1 and 3 years. Additional barium swallows, pathophysiological studies, and endoscopies were performed whenever patients reported recurrent GERD symptoms or the onset of new symptoms. Patients with BE entered the follow-up protocol recommended by international guidelines.^20^

### Clinical assessment after 20 years

At follow-up, patients were interviewed, their weight and height were recorded, along with the use of PPI prescribed for typical GERD symptoms (this parameter was recorded as ‘yes’ if PPI were used continuously or for more than 4 weeks). The initially-used SS was administered again, and patients were asked to quantify their satisfaction with their LARS on a scale from 0 to 10 (0 = completely unsatisfied, 10 = completely satisfied). Patients’ willingness to have surgery again was recorded on a scale from 0 to 2 (0 = I would not have surgery again, 1 = not sure, 2 = I would have surgery again). Patients were also asked to undergo endoscopy, barium swallow, high-resolution esophageal manometry (HRM) and 24-hour pH-monitoring. HRM was performed with a catheter 4.2 mm in diameter with 36 solid-state circumferential sensors spaced at 1-cm intervals and spanning the whole esophagus (Medtronic, Minneapolis, MN, USA). The manometric data were analyzed using ManoViewTM software (Medtronic). The HRM protocol has been described elsewhere.^21^ Barium swallow and 24-hour pH-monitoring were performed in the same way as in the preoperative work-up.

### Definition of surgical failure

LARS was judged to have failed in any of the following cases:

1. GERD symptom recurrence (SS >10, i.e.: the 10^th^ percentile of preoperative symptoms calculated on the patient population as a whole);
2. recurrence (or persistence) of Grade B or higher reflux esophagitis identified on endoscopy;
3. HH recurrence or slipped fundoplication (even in asymptomatic patients, if noted on barium swallow or endoscopy);
4. pathological 24-hour pH-monitoring (DeMeester score >14.72), even in asymptomatic patients;
5. Postoperative onset of dysphagia (redo-fundoplication or endoscopic dilation)
6. BE progression or onset of esophageal adenocarcinoma

### Statistical analysis

Numerical data were summarized as medians and interquartile ranges (IQR), and categorical data as absolute frequencies (n) and relative frequencies (%). Continuous variables were compared with Student’s t-test or the Mann-Whitney test, and categorical variables with the χ^2^ or Fisher’s exact test, as appropriate. Survival curves were estimated using the Kaplan-Meier method and compared using the log-rank test. A p-value lower than 0.05 was considered significant. The statistical analysis was run using R 4.1 software (R Foundation for Statistical Computing, Vienna, Austria).

## Results

**Fig. 1.**
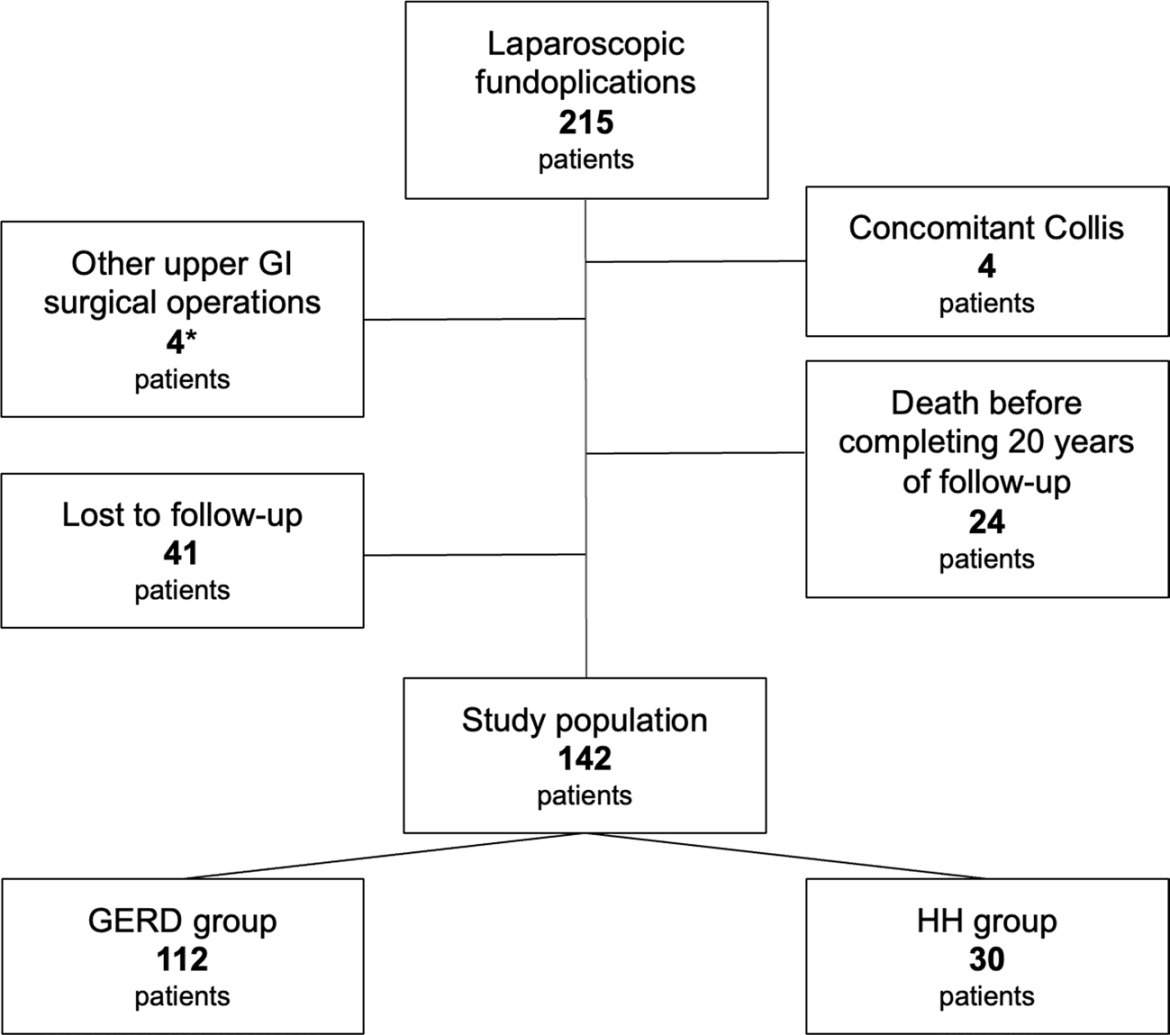
Study population flowchart. *One surgical operation for aorto-mesenteric compass syndrome, three sleeve gastrectomy.

Between 1992 and 2001, 215 patients underwent LARS at our department. Four patients (1.8%) had concomitant Collis gastroplasty and were excluded. During the follow-up, 4 patients (1.8%) had other upper gastrointestinal surgery (1 surgical operation for aorto-mesenteric compass syndrome, 3 sleeve gastrectomy), therefore they met exclusion criteria. Before completing 20 years of follow-up, 24 patients (11.2%) died for reasons not attributable to the LARS, while 41 patients (19%) were lost to follow-up (Fig. 1). The study population thus consisted of 142 patients: 112 in the GERD group and 30 in the HH group. Patients’ characteristics and details of their surgical procedures are listed in Table 1 and Table 2, respectively. Conversion to open surgery proved necessary in 8 patients (5.6%), and intraoperative and perioperative complications were recorded in 9 (6.3%) (Table 3).

**Table 1.** Characteristics of patients in the two groups

	GERD group (*n*=112)	HH group (*n*=30)	*p*-value
Female/Male	42/70	23/7	0.0003
Age (years) ^a^	46 (34-55)	64 (55-68)	<0.0001
BMI (kg/m^2^) ^a^	25.6 (23.5-28.1)	27.6 (25.3-28.9)	0.03
Symptom score ^a^	15 (9-19)	14 (8-21)	0.74
Symptom duration (months) ^a^	30 (12-60)	36 (12-60)	0.79
Basal LES pressure (mmHg) ^a^	8 (5-11)	10 (6.5-14.5)	0.09
LES total length (mm) ^a^	35 (28-43)	32 (22.5-36)	0.09
LES abdominal length (mm) ^a^	22 (17-31)	20 (11-24)	0.02
DM score ^a^	32 (19-51)	21 (10-60)	0.37
Esophagitis	83 (74.1)	18 (64)	0.23
BE	17 (15.2)	2 (6.7)	0.36
Hiatal hernia			<0.001
Type I	61 (55)	0	
Type II	0	4 (13.3)	
Type III	0	25 (83.3)	
Type IV	0	1 (3.3)	

BMI = body mass index; LES = lower esophageal sphincter; DM score = DeMeester score. Data expressed as n (%) or as ^a^ median (IQR); BE = Barrett’s esophagus

**Table 2.** Characteristics of LARS in the two groups

	GERD group (*n*=112)	HH group (*n*=30)	*p*-value
Fundoplication			0.47
Nissen	104 (93%)	26 (87%)	
Toupet	8 (7%)	4 (13%)	
Duration of surgery (minutes) ^a^	153 (120-180)	190 (145-241)	0.01
Conversion to laparotomy	0 (0)	8 (27%)	<0.0001
Mesh	0 (0)	11 (37%)	<0.0001
Hospital stay (days) ^a^	4 (3-5)	4 (4-6)	0.09

Data expressed as n (%) or as ^a^ median (IQR).

**Table 3.** Intraoperative and postoperative complications of LARS

	GERD group (*n*=112)	HH group (*n*=30)	*p*-value
Intraoperative			
gastric lesion	1 (0.9)	0	>0.99
Postoperative			
pneumonia	2 (1.8)	1 (3.3)	0.51
pneumothorax	2 (1.8)	0	>0.99
atrial fibrillation	1 (0.9)	0	>0.99
trocar-related abdominal wall hematoma	0	1 (3.3)	0.21
ureteral colic	1 (0.9)	0	>0.99
Total	7 (6.3)	2 (6.6)	>0.99

Data expressed as n (%)

**Fig. 2.**
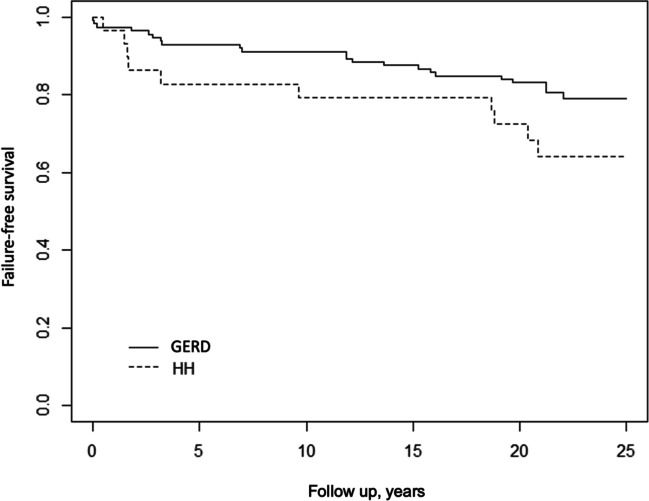
Kaplan-Meier curves showing the failure-free survival over time after LARS in the GERD and HH groups

At a median follow-up of 22 years (IQR: 21-24), the success rate in the study population was 76.8% (109/142): 80.3% (90/112) in the GERD group, and 63.3% (19/30) in the HH group (*p*=0.09). The Kaplan-Meier curves for the two groups are shown in Fig. 2 (GERD vs. HH *p*=0.07).

Gas bloat syndrome developed in 6 patients (4.2%): 5 in the GERD group, and 1 in the HH group. Revisional surgery was necessary in 14 patients (9.8%): 9 in the GERD group, and 5 in the HH group. This was due to an early dysphagia in 5 patients (conversion of Nissen to Toupet), a slipped fundoplication or hernia recurrence in 6 cases, to pH-detected abnormal acid reflux and esophagitis resistant to medical therapy in 2 cases, and to herniation of the stomach inside the fundoplication (telescoping) in one.

After 20 years of follow-up, an overall 86.8% of patients were satisfied or very satisfied with their LARS.

### GERD group

Median age, preoperative BMI, score and duration of symptoms, and manometric values are shown on Table 1. Preoperatively, all patients had an abnormal acid exposure of the distal esophagus on 24-hour pH monitoring. while esophagitis was endoscopically detected in 83 cases (74.1%).

The indications for surgery were typical symptoms with or without esophagitis in 78% of patients, atypical symptoms in 4%, Barrett’s esophagus in 17%, and peptic stenosis in 1%. The characteristics of LARS are shown on Table 2. A hiatoplasty according to Allison’s technique was performed in almost all patients. No meshes were applied and the operation was completed laparoscopically in all patients. Intraoperative complications included one (0.9%) gastric lesion, that was repaired intraoperatively without further consequences. Postoperative complications are summarized in Table 3.

After a follow-up of at least 20 years (median 22 years, IQR: 21-24), a successful outcome was confirmed in 90/112 patients (80.4%), and surgical failures were recorded in 22 (19.6%). Intrathoracic wrap was the most frequent type of failure in the GERD group (8/22, 36.4%). However, revisional surgery due to symptoms recurrence was necessary in 4 of these patients (18.2%), the remainder had a radiological recurrence with no clinical correlation. Five of 22 patients (22.7%) failed due to post-operative onset of dysphagia: all of these patients underwent Nissen fundoplication and required the dismantle of the fundoplication with conversion to Toupet fundoplication. Nine additional patients (40.9%) failed due to one or more of the above-mentioned causes of failure. These patients were managed by resuming PPI treatment.

**Fig. 3.**
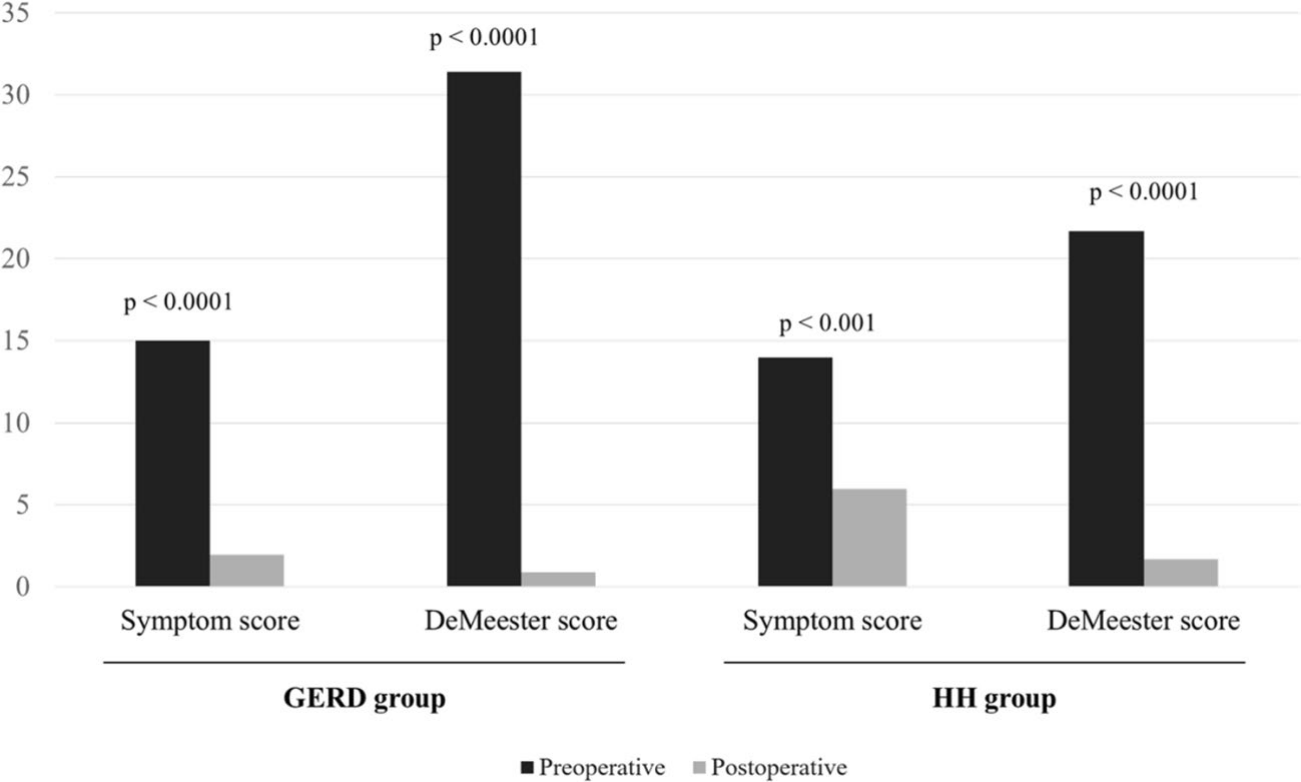
Subset analysis on: (1) pre- and postoperative symptom score; and (2) pre- and postoperative DeMeester score in the GERD and HH groups

Good symptom control was recorded in 93.5% of patients, and complete remission of heartburn and acid regurgitation was achieved in 58%. On 24-hour pH monitoring, the DeMeester scores significantly decreased (Fig. 3).

Medical therapy with PPI was resumed by 30/112 patients (26.8%), and surgery had failed in 13 of them.

**Fig. 4.**
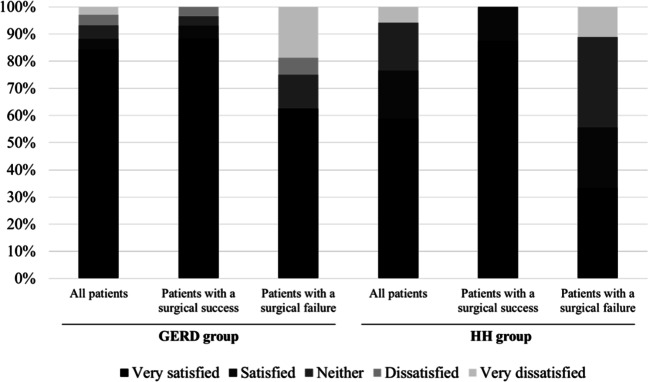
Patient satisfaction in the GERD and HH groups

**Fig. 5.**
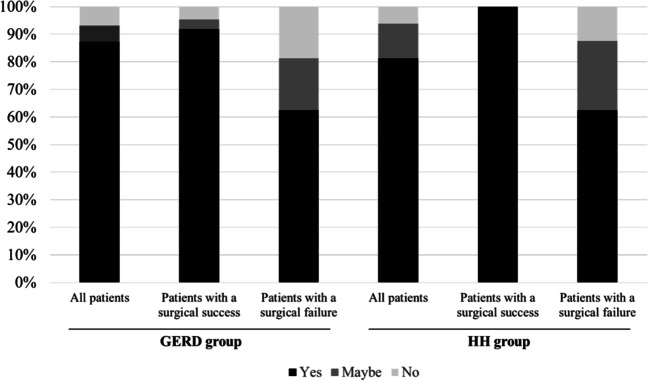
Patients’ willingness to undergo surgery again in the GERD and HH group

As for patients’ satisfaction more than 20 years after their surgery, 87.6% reported being satisfied, and only 2.8% were very dissatisfied (Fig. 4). Asked whether they regretted their decision to undergo surgery, 86.7% of patients said they would make the same decision to have surgery, while 6.7% would not (Fig. 5). As expected, patients whose LARS procedure failed were less likely to say they would have surgery again (*p*=0.005), and less satisfied (*p*=0.006).

At univariate analysis, no statistically significant association emerged between patients’ preoperative clinical, endoscopic and functional characteristics, and their outcomes (Table 4).

**Table 4.** Univariate analysis: predictors of failure of LARS in the GERD group

*p*-value	Outcome		
	Failure (*n*=22)	Success (*n*=90)	Variable
0.71	8/14	35/55	Female/Male
0.60	(23.6-28.1) 26.7	25.5 (23.5-28.1)	BMI (kg/m2)^a^
0.53	(1.1-2.7-) 1.5	(1.0-2.1-) 0.5	BMI variation^a^
0.14	(11-21) 18	(8-19) 14	Pre-op. symptom score^a^
0.10	(12-36) 18	30 (17-60)	Symptom duration (months)^a^
0.06	16(94%)	66(73%)	Esophagitis^a^
0.37	10	44	Grade A-B
	4	8	Grade C-D
	2	14	BE
0.08	(1-2) 2	(1-2) 2	Hiatoplasty (stitches)^a^
0.63	(5-10) 7	8 (6-11)	Basal LES pressure (mmHg)^a^
0.79	(25-48) 36	34 (28-43)	LES total length (mm)^a^
0.98	(16-35) 25	22 (17-30)	LES abdominal length (mm)^a^
0.72	(15-55) 39	33 (20-49)	DM score^a^
0.88	(5.1-14.9) 9.1	8.2 (4.8-12.4)	TOT ^a^%
0.61	(3.4-11.8) 7.7	7.1 (2.6-10.2)	ER ^a^%
0.92	(1.2-18.7) 5.6	8.0 (3.7-16.6)	SUP ^a^%
0.80	(32-75) 42	49 (28-69)	N.RGE^a^
0.38	(2-8) 3	5 (3-8)	5min ^a^<
0.44	(6-47) 22	25 (15-38)	LONG^a^

BMI = body mass index; LES = lower esophageal sphincter; BE = Barrett’s esophagus; DM score = DeMeester score; %TOT = % total esophageal exposure to acid; %ER = % of time of esophageal exposure to acid while upright; %SUP = % of time of esophageal exposure to acid while supine; N.RGE = number of acid refluxes; >5min = number of refluxes lasting more than 5 minutes; LONG = duration (in minutes) of the longest reflux. Data expressed as n (%) or as ^a^ median (IQR)

### HH group

Clinical characteristics are shown on Table 1. Preoperative endoscopy showed reflux esophagitis in 18 patients (60%). Barium swallow revealed a type III HH in most of these patients (83.3%). On preoperative 24-hour pH-monitoring, only half of the patients showed abnormal exposition to acid in the distal esophagus, with an overall median value of 20.85 (IQR: 10.2-60.5).

The characteristics of LARS are shown on Table 2. Hiatoplasty according to Allison’s technique was required in all cases, a non-absorbable mesh was also applied in 11 subjects (36.7%) for an evident tension in the hiatoplasty (2 cases), presence of an upside-down stomach (6 cases) or a perceived high risk of recurrence due to crura weakness (3 cases). Conversion to open surgery was necessary in 8 patients (26.7%) due to the difficulty of reducing the hernia safely. There was no intraoperative mortality or morbidity. Postoperative complications are summarized in Table 3.

At a 22-year median follow-up (IQR: 21-23), surgery had been successful in 19/30 patients (63.3%), and failed in 11 (36.7%). The unsuccessful procedures concerned 5/11 patients whose surgery had included the application of a mesh (45.5%), and 6/19 patients treated without applying a mesh (31.6%, p = n.s.).

Medical therapy with PPI was resumed by 7/30 patients (23.3%), and surgery had failed in 6 of them.

Five of the 11 patients whose surgical procedure failed (45.5%) required revisional surgery: 4 of them had a slipped fundoplication, and 1 had GERD identified on 24-hour pH monitoring. A laparoscopic Nissen fundoplication was performed in 4 patients, and an open Nissen fundoplication in 1. Four additional patients (4/11, 36.4%) showed radiological evidence of hernia recurrence with no clinical correlation, revisional surgery was therefore deemed unnecessary. Two additional patients (18.2%) failed due to a combination of the above-mentioned causes. These subjects were treated with medical therapy.

Twenty or more years after LARS, 76.7% of patients were completely satisfied with the outcome, and only 6.7% were very unsatisfied (Fig. 4). Asked whether they regretted their decision to undergo surgery, most patients (81.2%) said they would opt for surgery again (Fig. 5).

The univariate analysis showed that the preoperative SS was significantly higher among patients whose surgical procedure failed. There was also a trend towards a statistically significant difference in the duration of their symptoms before surgery (*p*=0.09), and their preoperative basal LES pressure (*p*=0.08) (Table 5).

**Table 5.** Univariate analysis: predictors of failure of LARS in the HH group

*p*-value	Outcome		
	Failure (*n*=11)	Success (*n*=19)	Variable
0.99	10/1	13/6	Female/Male
0.39	26.6 (24.6-28.5)	27.7 (26.4-29.2)	BMI (kg/m2)^a^
0.27	0.0 (1.2-1.1)	1.4 (3.2-0.0)	BMI variation^a^
0.02	21 (16-22)	12 (7-18)	Pre-op. symptom score^a^
0.09	19 (12-33)	60 (24-60)	Symptom duration (months)^a^
0.26	5 (45.5)	13(68.4)	Esophagitis^a^
0.78			Hiatal hernia
	2 (18.2)	2 (10.5)	Type II
	9 (81.8)	17 (89.5)	Type III
	0	0	Type IV
0.96	3 (4-3)	3 (4-3)	Hiatoplasty (stitches)^a^
0.71	5(45.5)	6 (32)	Mesh
0.08	9 (6-11)	12 (7-18)	Basal LES pressure (mmHg)^a^
0.27	34 (28-41)	29 (22-34)	LES total length (mm)^a^
0.71	21 (11-27)	20 (12-21)	Abdominal total length (mm) ^a^
0.54	20 (9-40)	32 (14-73)	DM score ^a^
0.40	3.6 (2.9-9.0)	8.7 (2.9-17.4)	TOT ^a^%
0.40	1.7 (1.0-5.1)	2.3 (1.7-6.2)	ER ^a^%
0.45	4.3 (0.4-7.7)	6.6 (2.9-26.0)	SUP ^a^%
0.65	30 (25-67)	29 (12-69)	N.RGE ^a^
0.79	2 (1-6)	2 (1-7)	5min ^a^<
0.43	18 (9-30)	31 (8-47)	LONG ^a^

BMI = body mass index; LES = lower esophageal sphincter; DM score = DeMeester score; %TOT = % total esophageal exposure to acid; %ER = % of time of esophageal exposure to acid while upright; %SUP = % of time of esophageal exposure to acid while supine N.RGE = number of acid refluxes; >5min = number of refluxes lasting more than 5 minutes. LONG = duration (in minutes) of the longest reflux. Data expressed as n (%) or as ^a^ median (IQR).

## Discussion

Thirty years after the noninvasive approach to antireflux surgery was introduced, laparoscopic fundoplication represents the gold standard among surgical treatments for patients with GERD. However, information about how long the efficacy of LARS lasts is still limited. Several published papers present short-term assessments on the results of laparoscopic fundoplication, while only a handful report on a follow-up beyond 15 years.^22–25^

The present study concerns one of the largest populations to have been followed up for more than 20 years and indicates a success rate of 80.4% among uncomplicated GERD patients and 63.3% among patients with large HH. Our data confirm that patients who have LARS for GERD and those who undergo the procedure for large HH should be considered separately because these two populations have different demographic and clinical characteristics, and technical issues as well.

There is no consensus on the definition of a failed surgical antireflux procedure for uncomplicated GERD. Some studies mainly consider subjective symptom recurrence, others focus on quality of life, or postoperative complications, or patients’ satisfaction, objectively-demonstrable recurrent GERD, or the resumption of medical treatment. Robinson et al. retrospectively examined data collected on satisfaction, PPI use, and symptoms from 51 patients who underwent LARS for uncomplicated GERD, and had a median follow-up of 19.7 years. They did not consider patients with giant HH.^22^ They found that 90% of patients reported being satisfied with the treatment, and 75% had a complete resolution of typical GERD symptoms. On the other hand, 43% had to resume PPI treatment after the fundoplication, and 17.6% of patients underwent surgical revision. Sadowitz et al considered 27 patients a median 19 years after laparoscopic fundoplication for GERD. They used patient satisfaction to assess final outcome, and reported that 95% of patients would have had the same operation again, and 88% of patients were satisfied or very satisfied.^23^ Csendes et al. analyzed the results of LARS 15 years after the procedure using a symptom score (Visick III-IV) as a criterion to identify surgical failures.^24^ They found that laparoscopic fundoplication for GERD had been successful in 80% of patients. One of the few published studies to have analyzed the long-term results using objective diagnostic criteria was conducted by Salminem et al.^25^ The authors endoscopically examined 36 patients ten years after they had underwent LARS for GERD, finding a defective plication in 11.1% of patients. In 5.4% of cases in their study, patients had subsequently undergone revisional surgery - a reoperation rate comparable with ours.

Although they are important indicators when dealing with a functional disease, focusing only on patients’ symptoms, satisfaction, and/or use of PPI cannot suffice as a definitive indicator of the outcome of LARS because such criteria are subjective. That is why we expanded our definition of surgical failure to include objective (radiological, endoscopic, and pH-metric) parameters to assess the durability of LARS more accurately. Our data thus confirm the findings of well-conducted studies with a shorter follow-up,^26^ and also show that the surgical procedure is a valid, long-term treatment option for carefully-selected patients with uncomplicated GERD, as an alternative to the continuous use of high-dose PPI therapy.

Experience gained with laparoscopic surgery, and its subsequent diffusion, led to the use of this approach in the treatment of more complex conditions, such as large or giant HH, which had required open surgery until the 1990s.^27^ The literature shows that LARS achieves similar results to open surgery in such situations, but using the laparoscopic approach in patients with giant HH is associated with a more variable failure rate than when LARS is used to treat patients with uncomplicated GERD.^28–30^ This is probably because the types of patient differ considerably, those with HH being older, with important comorbidities and associated therapies. Another possible explanation concerns the technical difficulties encountered during the procedure, such as the dimensions of the hiatus that needs to be closed without applying too much tension on the pillars, or the feasibility of completely removing the hernia sac.^31^ A study by Fei et al. found that the muscle tissue of the pillars in patients with giant HH presented some degenerative patterns.^32^ Such observations are confirmed by our data: patients with giant HH had a higher rate of conversion to open surgery, an older median age, and a lower success rate (63.3%) 20 years after LARS than patients with GERD.

The most common types of failure in patients treated with laparoscopic fundoplication for large HH concern hernia recurrence or slipped fundoplication. Hashemi et al. reported a 43% rate of radiological relapse in patients with type II hernias at a median follow-up of 17 months, although only 24% of the patients affected reported poor symptom control.^33^ This goes to show that radiological findings do not correlate with subjective symptoms in cases of hernia relapse, as already widely documented in the literature, and confirmed by our data too. In fact, 50% of our patients who underwent LARS for HH that later proved unsuccessful nonetheless reported being satisfied or very satisfied with the procedure.

At univariate analysis, the presence of esophagitis was higher in patients whose surgical treatment failed, even though this did not reach a statistical significance. Moreover, the severity of esophagitis and the presence of Barrett were not associated with a worse outcome in our cohort. Indeed, previous studies confirmed that the subjective and objective outcome of fundoplication at 5 years were similar in both erosive and non-erosive reflux disease.^10^ As for Barrett the results are also controversial: while some authors reported relatively poorer outcomes of anti-reflux surgery in patients with BE, other studies reported comparable postoperative improvement of symptoms among patients with or without BE.^34–36^

While only two GERD patients with preoperatively-detected BE failed in our cohort, two additional patients in this group developed an adenocarcinoma postoperatively, even though no BE was present at the index endoscopy. Taken together these findings support the need for a strict postoperative surveillance, especially for patients with preoperative severe esophagitis or BE.

The use of a mesh reinforcement during surgery for HH remains a controversial topic. The most debated aspects concern hernia recurrences and the risk of the mesh penetrating the esophageal wall. There was 1 mesh-related complication among all patients who underwent LARS at our department between 1992 and 2001, consisting in the mesh migration into the esophagus, which necessitated esophagectomy and intrathoracic esophagogastroplasty (as in other cases reported in the literature^37–39^). However, this patient died from mesothelioma before completing 20 years of follow-up, therefore was excluded from our study. Among patients who underwent LARS for large HH, there was no association at univariate analysis between the use of a mesh and incidence of failure of surgical procedure (*p*=0.71). A recent systematic review of randomized controlled trials on mesh application vs. primary crural closure alone, conducted by Angeramo et al., identified a comparable long-term recurrence rate for the two approaches.^40^ Oelschlager et al. reported the results of a randomized trial of laparoscopic paraesophageal hernia repair comparing primary diaphragm repair with primary repair buttressed with a biologic prosthesis; at a median follow-up of 48 months, they found no differences either regarding complications and outcomes in the two groups, with a HH recurrence rate of more than 50%.^41^ These findings confirm that there is still no clear evidence to support the routine use of a mesh to reduce the risk of recurrence after HH surgery.^42,43^

Our study has some limitations to bear in mind. One concerns its retrospective design. Another lies in that (as explained earlier) our case series includes patients who underwent LARS when our department’s experience with the technique was limited, and surgeons were possibly still completing their learning curve. Changes over time in the technologies, materials and techniques available should also be considered when comparing our results with contemporary case series that have a shorter follow-up. The choice of the type of fundoplication (Toupet vs. Nissen), and any use of a mesh were also largely operator-dependent. Only two thirds of the patients agreed to undergo functional test during the follow-up (manometry and 24-hour pH monitoring). Albeit this may represent a selection bias, we think however the number is big enough to have a good post-operative picture of esophageal acid exposure in our patients. Finally, about 20% of our patients were lost to follow-up, and the relatively high drop-out rate for the postoperative diagnostic tests we proposed was related, at least in part, to the ongoing COVID-19 pandemic.

## Conclusion

Our study demonstrated that laparoscopic antireflux surgery is effective and durable (for >20 years) in patients with uncomplicated GERD and, to a lesser extent, in those with a large hiatal hernia.
